# Study on the Aging Behavior of an Ultra-High Molecular Weight Polyethylene Fiber Barrier Net in a Marine Environment

**DOI:** 10.3390/ma15165599

**Published:** 2022-08-15

**Authors:** Wangxuan Zhang, Xiaofei Jing, Yanqiang Bai, Xiaoming Shan, Xiaoyu Qi, Maoxin Yan, Zhongyu Cui

**Affiliations:** 1 China Nuclear Power Technology Research Institute Co., Ltd., Shenzhen 518124, China; 2 Daya Bay Nuclear Power Operations and Management Co., Ltd., Shenzhen 518124, China; 3 School of Materials Science and Engineering, Ocean University of China, Qingdao 266100, China

**Keywords:** ultra-high molecular weight polyethylene, ultraviolet, salt spray, hygrothermal, environmental spectrum

## Abstract

In the present work, the performance of ultra-high molecular weight polyethylene (UHMWPE) barrier nets in marine environments is investigated by Fourier transform infrared spectroscopy, thermogravimetry, scanning electron microscopy, and tensile experiments. The chemical, morphological, thermal stability, and strength changes after aging in salt spray, hygrothermal, and ultraviolet (UV) environments are characterized. An environmental spectrum is designed to simulate a real service environment and predict the service life of UHMWPE. The results show that UV energy can activate UHMWPE molecules and lead to chain breaking, which lowers the breaking strength more efficiently than salt spray. In a hygrothermal environment, the UHMPE fibers bond into clumps, which causes a slight increase in breaking strength after the initial rapid decrease with aging time. The acceleration ratio of the environmental spectrum increases with increasing aging time, which may be caused by the cross-linking and degradation of macromolecular chains in the material. The environmental spectrum given by this work can be used to evaluate performance and predict the service life of UHMWPE barrier nets in marine environments.

## 1. Introduction

Owing to its high strength, superior wear resistance, and chemical stability, ultra-high molecular weight polyethylene (UHMWPE) has been used as barrier to prevent the clogging of cold source intakes of nuclear power plants by marine organisms [1,2,3,4,5]. Even if fiber materials have been widely used in military and civil applications, degradation is inevitable during the process of storage, transportation, and service [6,7,8,9]. Aging behavior can also occur on UHMWPE fibers because of the interplay between internal and environmental factors, such as light, heat, oxygen, chemical, and biological erosion, leading to the deterioration in performance characteristics [10,11]. Therefore, it is important to evaluate the aging resistance of materials in a practical working environment. 

Environmental exposure tests and laboratory-accelerated aging tests such as hygrothermal and ultraviolet irradiation (UV) are used to evaluate the performance deterioration of UHMWPE [12,13,14,15]. Huang et al. [16] investigated the structural changes of high-density polyethylene (HDPE) after the application of stress and photo-oxidative aging experiments. The materials were more prone to degradation and more susceptible to molecular chain scission and chain break under the aging process, rendering the flaws to appear on the surface of the sample. Gulmine et al. [10] used UV- and xenon arc radiation tests to simulate ageing of different kinds of PE including low-density polyethylene (LDPE), linear low-density polyethylene (LLDPE), and HDPE. After aging with different exposure times and different temperature cycles, polar groups, chain breaking, and cross-linking may occur in PE.

In a nuclear power plant, UHMWPE is used as a barrier net to prevent the ingress of plankton and ensure cleaning of the cooling water from the ocean. In this case, the barrier net is exposed to the marine environment and to salt spray aging processes. In this paper, UV irradiation, hygrothermal, and salt spray aging tests are used to simulate the operating environment of UHMWPE in a tropical marine environment in China. Surface morphologies, fracture strength, and chemical variations are determined. Subsequently, an environmental spectrum is established to simulate the real service environment considering all of the factors, and the accelerated ratio of the environmental spectrum is evaluated.

## 2. Experimental

### 2.1. Materials

**Figure 1:**
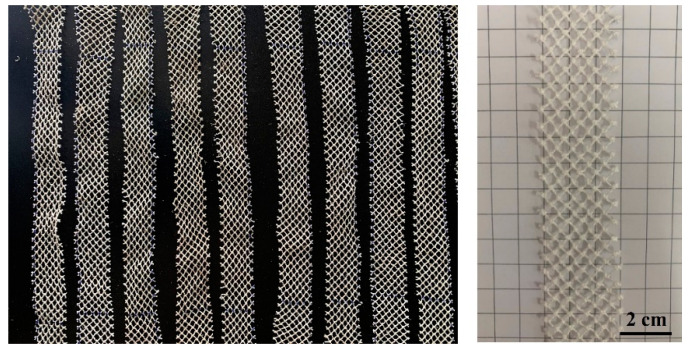
Macro-morphologies of the UHMWPE samples used for the ageing and tensile tests.

The material used in this study was ultra-high molecular weight polyethylene (UHMWPE) with a wire diameter of 0.6 mm and a mesh diameter of 3.5–4.0 mm. The UHMWPE barrier net was woven into a network through knots by several fine fibers, which were twisted into a strand. Samples with a length of 300 mm and a width of 4.5 mesh were taken from an as-received barrier net for acceleration aging tests in a laboratory, as shown in Figure 1. Barrier nets that have been used in marine for several months were also evaluated.

### 2.2. Aging Tests

#### 2.2.1. Ultraviolet Aging Experiment

The UV aging test equipment was an UV weather test box with a type 1A (UVA-351) lamp, supplied by Shanghai Forest Instrument Co., LTD. The wavelength of UV radiation was 340 nm, and the intensity was 0.76 W/m^2^. For the UV test, the temperature was set to 60 °C, the samples were irradiated for 24 h per cycle, and a total of 10 cycles were performed. 

#### 2.2.2. Salt Spray Aging Experiment

The salt spray test equipment was conducted with the LRHS-412-RFHY type test chamber of Forest Frequency Company. The samples were sprayed by 5 wt.% NaCl at 35 °C according to ISO4611-2010. Each cycle lasted for 10 days and the longest exposure time was 60 days of six cycles. 

#### 2.2.3. Hygrothermal Aging Experiment

The LHS-100CL type test chamber was used to perform the hygrothermal experiment. The experiment condition was set to 50 °C and 93% humidity, and the conductivity of the water vapor used to maintain the humidity of the test chamber was less than 20 μS/cm. For each cycle, the samples were exposed in the chamber for 10 days. The longest exposure time was 60 days of six cycles.

#### 2.2.4. Environmental Spectrum

**Figure 2:**
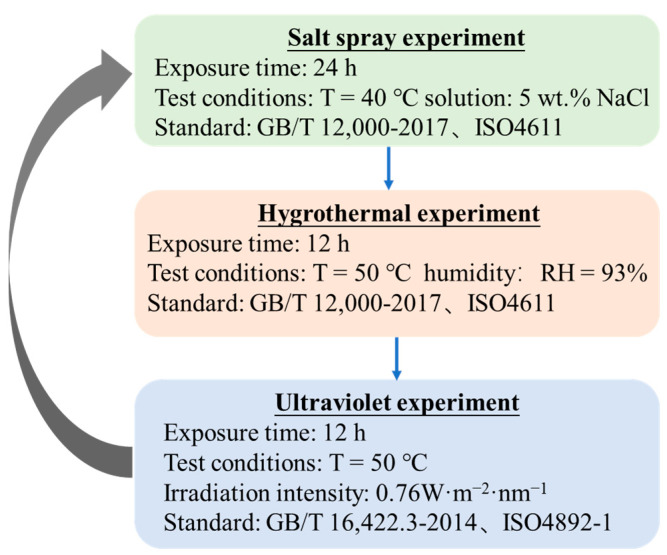
The environmental spectrum designed to simulate the real service environment of UHMWPE.

An environmental spectrum was designed to simulate the performance of the UHMWPE barrier net in the actual environment according to the ultraviolet, hygrothermal, and salt spray experimental results. The environmental spectrum was composed of ultraviolet, hygrothermal, and salt spray experiments, as shown in Figure 2. For each cycle, after being sprayed by 5 wt.% NaCl at 35 °C for 24 h, the samples were moved to the hygrothermal chambers, which were set to 50 °C and 93% humidity, for 12 h. Then, the samples were exposed to UV with an irradiation intensity of 0.76 W/m^2^ for another 12 h at 50 °C.

### 2.3. Analysis Methods

#### 2.3.1. Tensile Testing

**Figure 3:**
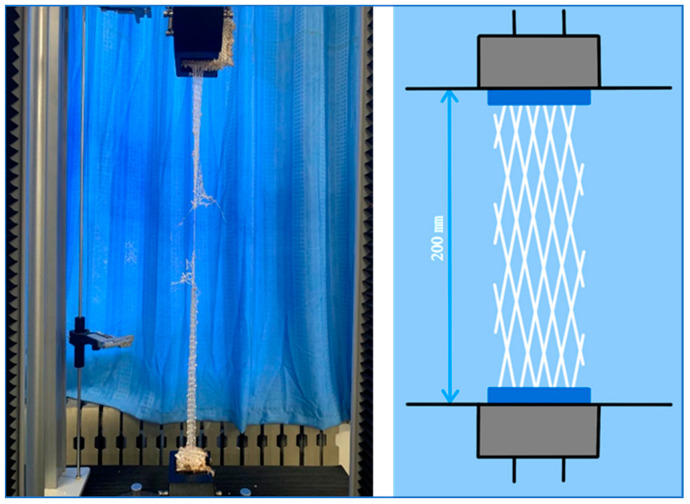
Tensile test of the ultra-high molecular weight polyethylene fiber interception net.

The tensile properties were characterized by a GOTECH tensile testing machine. According to Chinese standard GB/T4925-2008, the breaking strength of UHMWPE after natural aging and artificial aging was derived. As shown in Figure 3, the UHMWPE mesh was evenly clamped into the upper fixture, and the stretching rate was 500 mm/min. The material should be attached to the inside of the drawing machine fixture to avoid sample clamping or slippage. At least six duplicate samples were used to take average values. It should be noted that, if the sample was broken in the fixture, the measured value was invalid and abandoned. 

#### 2.3.2. Microstructure Analysis

The scanning electron microscope (ZEISS, Gemini-SEM 300, Oberkochen, Germany) was used to observe morphology of samples with a length of about 1 cm in the middle part of the mesh. The sample surfaces were coated with a layer of gold with a thickness of 8~10 nm to ensure the conductivity. The samples were attached to the sample stage with conductive adhesive. During the tests, the working distance (WD) of SEM was about 8.5 mm, the working voltage was 10 kV (EHT = 10 kV), and the signal mode was secondary electron mode.

#### 2.3.3. Infrared Spectral Analysis

Infrared spectral analysis was carried out in a Nicolet iS50 Fourier transform infrared spectrometer in attenuated total reflectance (ATR) mode because of the opacity of UHMWPE fibers. The laboratory temperature was kept at 20 °C and the relative humidity was below 65%. The number of scans was 32 and the resolution was 4 cm^−1^. It was tested in the wave-number range of 4000~400 cm^−1^. The changes in the fiber groups were judged according to the main absorption band and characteristic frequency in the spectra.

#### 2.3.4. TGA Measurement

Thermogravimetry analysis (TGA) was performed to observe the effect of the aging method on oxidation temperature and to determine the thermal stability with a TGA-Q5000 (TA Instruments, New Castle, DE, USA). The samples were heated from room temperature to 800 °C at a heating rate of 10 °C/min in a nitrogen atmosphere. 

## 3. Results

### 3.1. Infrared Spectra

**Figure 4:**
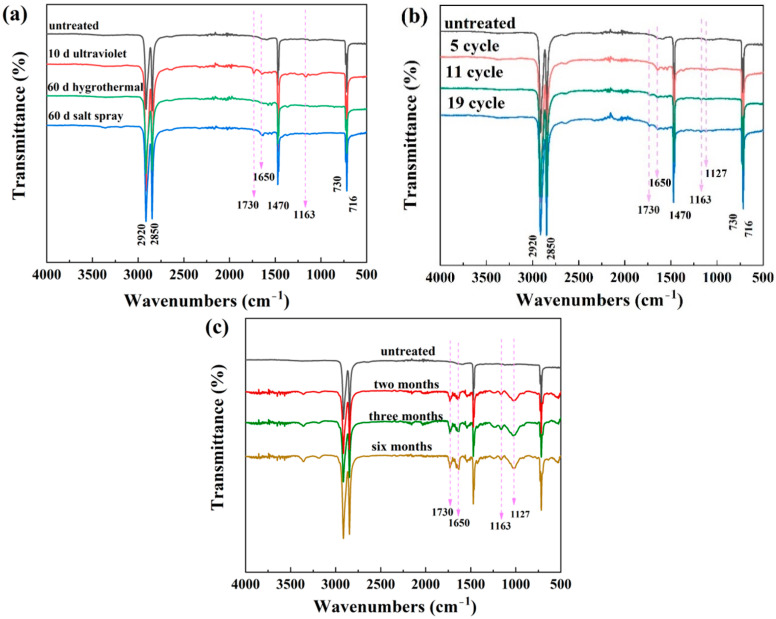
ATR-FTIR spectra of untreated and aged samples in individual salt spray, hygrothermal, and UV environments (**a**); the environmental spectrum (**b**); and the real service environment (**c**).

Aging can lead to long chain breaking and new side group formation of UHMWPE, and the changes in chain configuration and conformation can be reflected in infrared spectroscopy. Figure 4 shows the ATR-FTIR spectra of the UHMWPE samples after aging in different environments. The peaks at 2920 and 2850 cm^−1^ reflect the CH_2_ asymmetric stretching and stretching vibration. The peaks at 1470 and 1461 cm^−1^ are CH_2_ in-plane bending and wagging vibration of UHMWPE chains. The 730 and 716 cm^−1^ peaks belong to C-H out-of-plane bending vibration [17,18]. The positions of the above peaks in the ATR-FTIR spectra show little change after aging by comparison with those of the untreated samples.

Figure 4a shows that an absorption band around 1730 cm^−1^, which can be assigned to the C=O stretching vibration, and a band around 1163 cm^−1^ denoting C–O–C stretching vibration appears on the spectrum of UV irradiation [19,20]. The peak at 1650 cm^−1^ representing C=C stretching appears on the spectrum of both UV irradiation and salt spray. The three peaks appearing on the spectrum of UV irradiation also appear on the spectrum aging in the environmental spectrum for 19 cycles, as shown in Figure 4b, and they are even more obvious in the samples that are naturally aged in real environments, as observed in Figure 4c. The appearance of the above peaks is related to oxidation reactions of UHMWPE molecular, attributed to sunlight exposure and the presence of oxygen [16]. According to the free radical reaction mechanism, the increase in oxygen content can lead to a series of oxidation reactions under UV irradiation. The main chain of UHMWPE breaks and forms light-absorbing groups, which generate free radicals under the action of light, further induce oxidation reactions, and result in molecular degradation. Huang [21] reported that Gamma irradiation could result in oxidation on the surface of UHMWPE. During the irradiation process, free radicals generated in the amorphous and crystalline phase can react with oxygen to produce ketone groups in the presence of oxygen.

### 3.2. Surface Morphology

**Figure 5:**
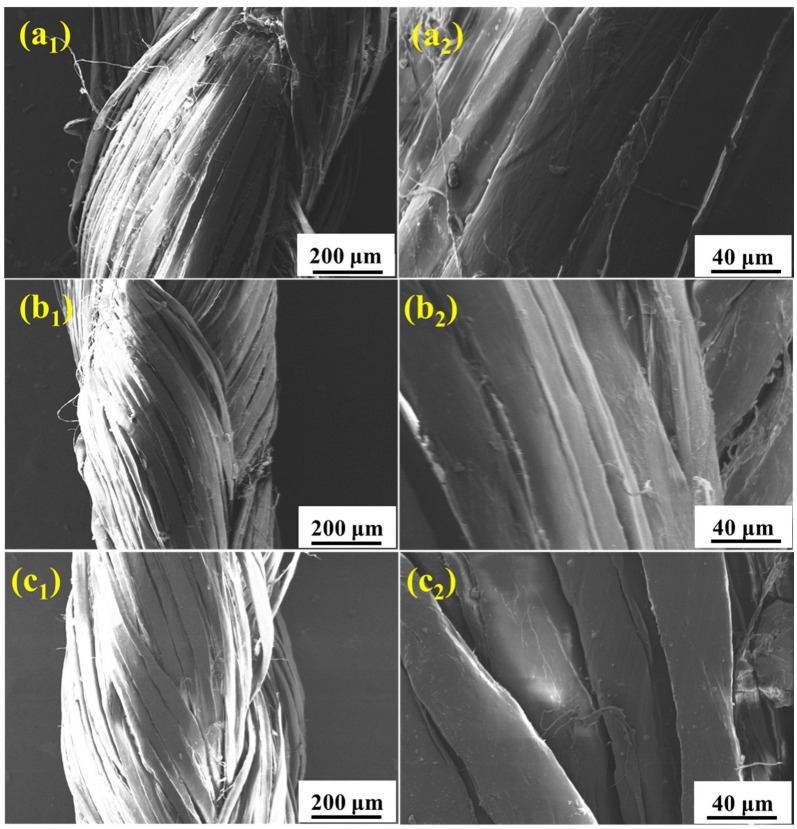
Surface morphologies of the UHMWPE fiber after aging in salt spray (**a_1_,a_2_**), hygrothermal (**b_1_,b_2_**), and ultraviolet (**c_1_,c_2_**) environments.

SEM is used to examine the surface morphology of the fibers and analyze the failure mechanism. Figure 5 shows the micro-morphology of the UHMWPE fiber surface after aging in salt spray, hypothermal, and ultraviolet environments, separately. After 60 days of salt spray aging (Figure 5(a_1_,a_2_)), the surface of the fiber is corroded. The existence of chloride ion with a small radius and high permeability in the salt spray environment results in the obvious shedding of the UHMWPE filament fiber. After 60 days of hygrothermal aging (Figure 5(b_1_,b_2_)), the surface of the UHMWPE fiber is no longer smooth, but no obvious surface damage is found in the high temperature and humid environment. Figure 5(c_1_,c_2_) shows that the fiber has obvious cracks and damage after UV irradiation for 10 days, which affects the mechanical properties of the material.

**Figure 6:**
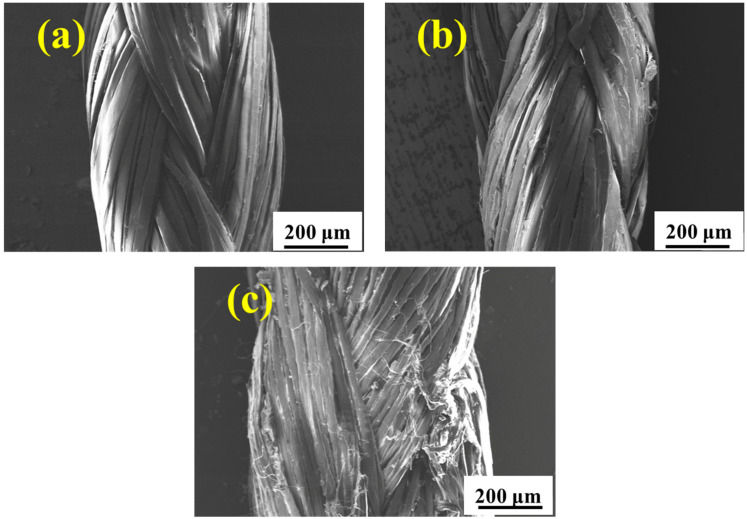
Surface morphologies of the UHMWPE fiber after aging in the environmental spectrum as described in Figure 2 for 5 cycles (**a**), 11 cycles (**b**), and 19 cycles (**c**).

The environmental spectrum as described in Figure 2 is designed to simulate the real service environment of the UHMWPE barrier net. Figure 6 shows the surface morphology of the UHMWPE fiber after aging in the environmental spectrum for different cycles. After five cycles (Figure 6a), no obvious damage occurs in the fiber surface. Upon prolonging the aging time to 11 cycles, slight abrasion is observed on the fiber surface, as shown in Figure 6b. Continuing to increase the aging time to 19 cycles, the abrasion in the fiber surface is more obvious, as illustrated in Figure 6c. 

**Figure 7:**
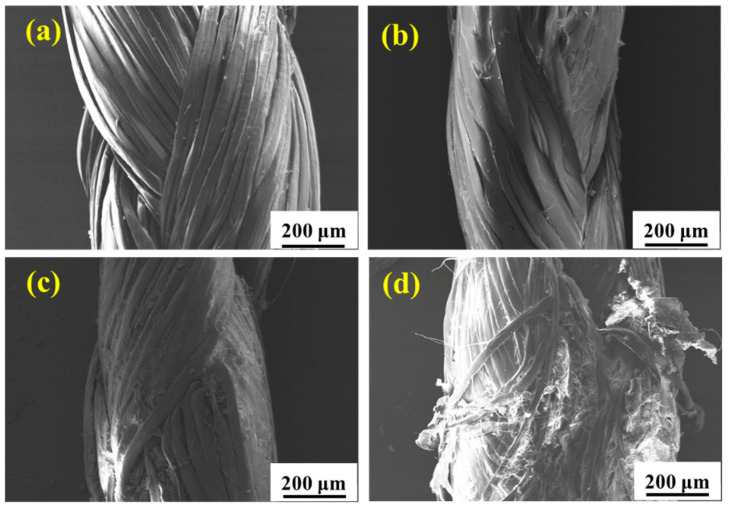
Surface morphologies of the UHMWPE fiber after aging in a real marine environment for 0 months (**a**), 2 months (**b**), 3 months (**c**), and 6 months (**d**).

Figure 7 shows the surface morphology of the UHMWPE fiber with different periods of service in the real marine environment. Figure 7a shows that the untreated fiber surface is initially smooth and unwrinkled. While after service, deterioration occurs and the surface of the fiber falls off significantly with the extension in service time owing to long-term UV irradiation and seawater immersion. The surface morphology of the UHMWPE fiber after aging in a real service environment for 6 months is similar with that in the environmental spectrum for 19 cycles, meaning that the environmental spectrum can simulate the real service environment, but more proof is needed.

### 3.3. Thermogravimetry Analysis (TGA)

**Figure 8:**
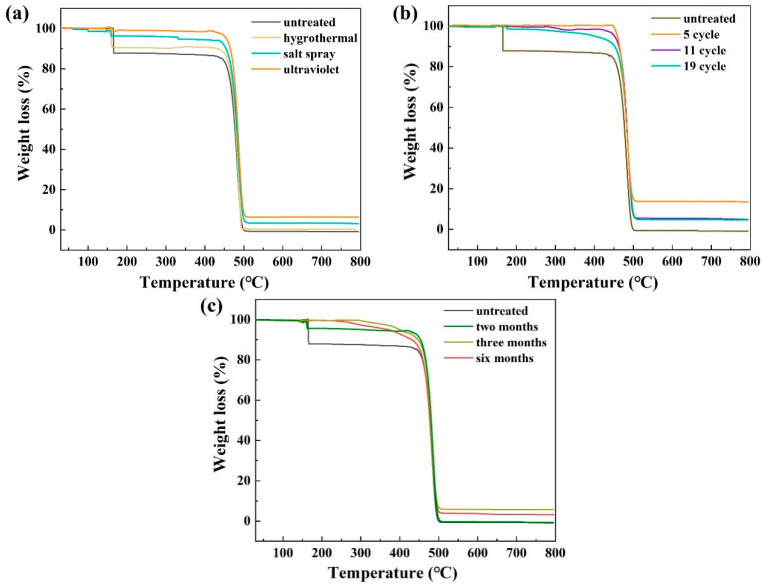
TGA curves of untreated and aged samples in separate salt spray, hygrothermal, and UV environments (**a**); the environmental spectrum (**b**); and the real service environment (**c**).

Figure 8 shows the TGA results of the UHMWPE fiber before and after aging. The specimen experiences two stages of weight loss within the measured temperature range. The first stage occurs at about 175 °C, where the most obvious weight loss occurs on the untreated samples. The weight loss at this stage mainly comes from volatilization of small molecules, such as water and cross-linked by-products. The moisture content of the untreated sample is apparently higher than that after aging, so its weight loss is more obvious in this stage. The samples after ultraviolet, salt spray, and hygrothermal treatment have different water contents, so their weight loss in this stage is different (Figure 8a). The second stage of weight loss occurs before 520 °C, where thermal decomposition of the UHMWPE main chain occurs and significant weight loss is observed [22,23]. The starting temperature of this stage can reflect the thermal stability of samples through different treatment. As shown in Figure 8a, no significant difference is observed by different aging methods separately. While after aging in the environmental spectrum and in outdoor exposure, thermal stability gradually decreases with the increase in aging cycles, as shown in Figure 8b,c, proving that the designed environmental spectrum is suitable for simulating the actual service environment of the UHMWPE barrier net. 

### 3.4. Mechanical Property and Fracture Morphology

**Figure 9:**
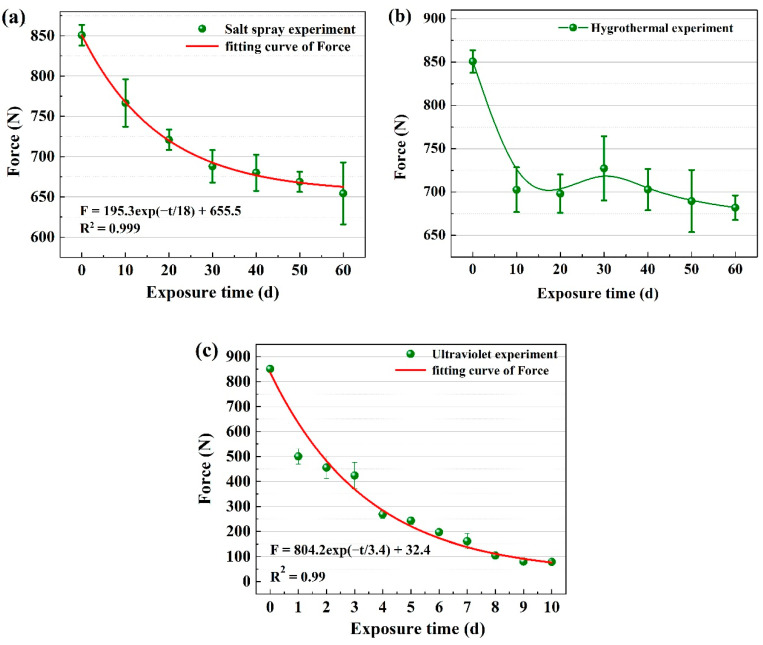
Breaking strength of the UHMWPE barrier net after aging in salt spray (**a**), hygrothermal (**b**), and ultraviolet (**c**) environments.

The deterioration in mechanical properties is one of the most evident effects of aging, which significantly affects the service safety and lowers the service life of UHMWPE. Figure 9 shows the mechanical property change after aging in salt spray, hygrothermal, and UV environments, separately. It is seen that the specimen without aging treatment has the highest breaking strength. In salt spray environments, the breaking strength of the UHMWPE fiber specimen decreases gradually with the increase in treatment time, and it decreases to 655 N after aging for 60 days (Figure 9a). In hygrothermal environments, the breaking strength experiences a rapid decrease from 850 N to 703 N after only 10 days of aging and then increases slightly to 725 N after aging for 30 days (Figure 9b). This is consistent with the findings of Luo et al. [24], who studied the moisture absorption behavior of UHMWPE composites and attributed the reinforcement of tensile strength to interactions of water and heat in the hygrothermal environment. On one hand, the movement of molecular chain segments is intensified by water and heat, and the molecular chain changes from a relatively disordered state to a regular arrangement, leading to agglomeration of fine fibers [25]. On the other hand, water molecules that invade the material act as internal plasticizers. In UV environments, the breaking strength decreases more rapidly than that treated in salt spray and hygrothermal environments (Figure 9c). According to the microstructure and infrared spectrum analysis, the oxidation degradation reaction occurs in the UHMWPE macromolecular chain under the condition of thermal and oxygen, and new groups such as the carbonyl group and carbon–carbon double bond appear [26]. With the extension in aging time, the oxidation degradation degree becomes increasingly serious, resulting in molecular chain fracture, molecular weight reduction, and a decline in tensile properties [27]. 

**Figure 10:**
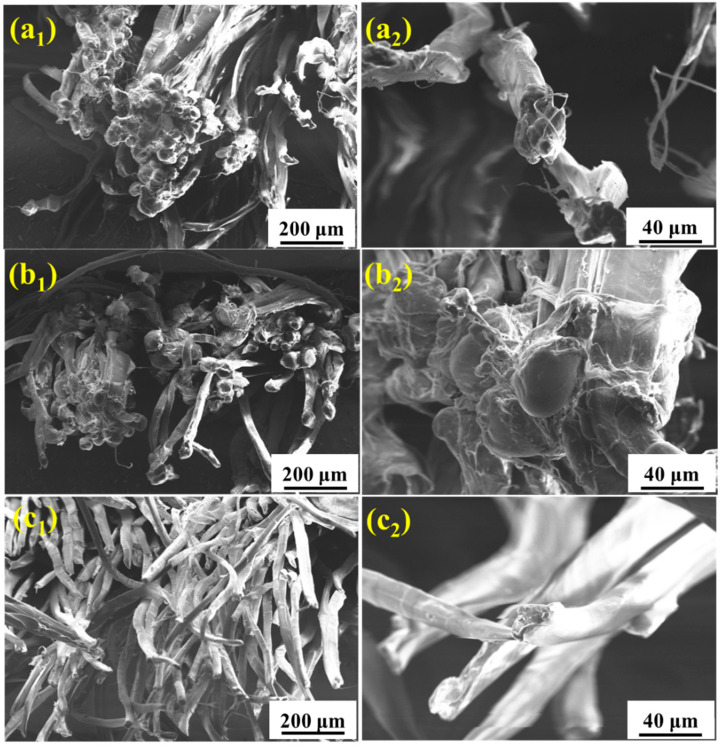
Fracture morphologies of the UHMWPE fiber after aging in salt spray (**a_1_**,**a_2_**), hygrothermal (**b_1_**,**b_2_**), and ultraviolet (**c_1_,c_2_**) environments for 60 days, 60 days, and 10 days, respectively.

**Figure 11:**
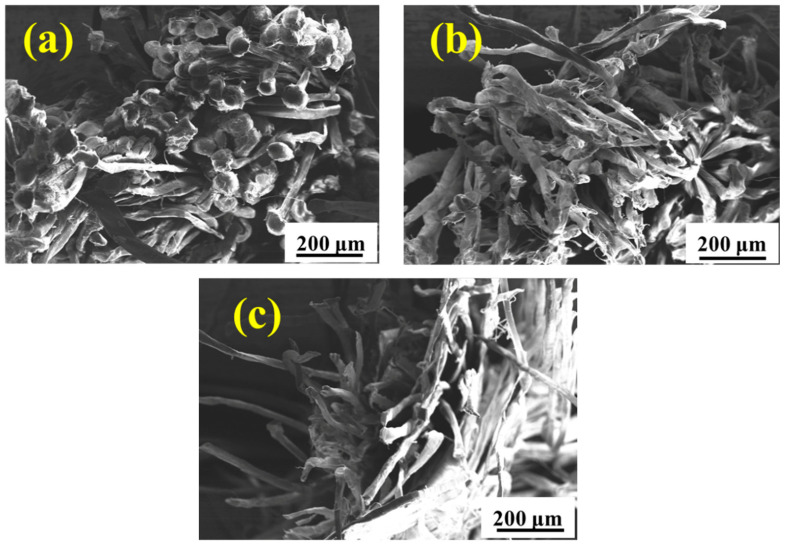
Fracture morphologies of the UHMWPE fiber after aging in the environment spectrum as described in Figure 2 for 5 cycles (**a**), 11 cycles (**b**), and 19 cycles (**c**).

**Figure 12:**
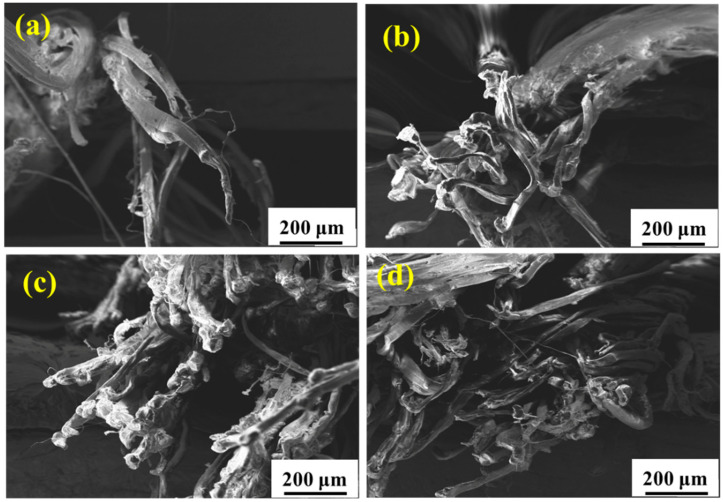
Fracture morphology of the UHMWPE fiber after aging in the real service environment for 0 months (**a**), 2 months (**b**), 3 months (**c**), and 6 months (**d**).

Figure 10 shows the fracture morphology of the tensile samples after aging in salt spray for 60 days, hygrothermal for 60 days, and UV for 10 days. It is seen that the failure of UHMWPE occurs in a ductile way, which is evident from the highly elongated debris of the failed surface [14,16]. After salt spray aging (Figure 10(a_1_,a_2_)), slight adhesion between thin fibers is observed. After hygrothermal aging (Figure 10(b_1_,b_2_)), several fine fibers are bonded into clumps, resulting in an increase in cross section, which slows down the loss of mechanical properties to a certain extent. After UV irradiation aging (Figure 10(c_1_,c_2_)), the fine fibers basically exist in the form of a single root without adhesion, and the fracture surface shows a neat brittle section, indicating a decrease in toughness [28]. Figure 11 and Figure 12 show the fracture morphology in the environmental spectrum and in real service environment, respectively. It can be found that brittle fracture of the port surface becomes obvious with the extension in aging time, indicating that the ultraviolet radiation is the main reason for the mechanical deterioration. The surface morphology and fracture morphology after outdoor service are consistent with that treated in the environmental spectrum, which proves that the environmental spectrum can reflect the actual service situation of UHMWPE by integrating humidity, heat, salt spray, and ultraviolet.

### 3.5. Accelerated Ratio of the Environmental Spectrum

**Figure 13:**
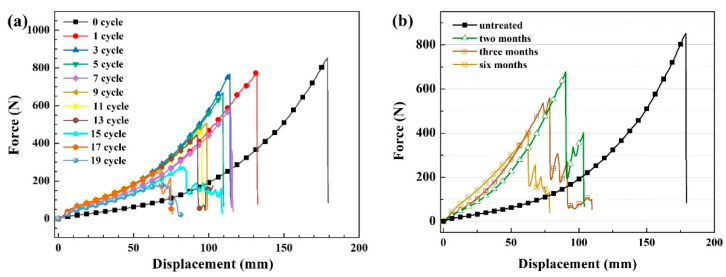
Tensile curves of the UHMWPE fiber interception net after aging in the environmental spectrum for different cycles (**a**) and the real service environment for different times (**b**).

Figure 13 shows the tensile curves of the UHMWPE fiber interception net in the designed environmental spectrum and in the real service environment. It is seen that the UHMWPE fiber after aging in the environmental spectrum and in the real service environment have the same elastic characteristics without yield point, and the fracture mode is brittle rupture. The breaking strength of the material decreases with the increase in aging time in both circumstances. After aging in the environmental spectrum for more than 15 cycles and in the real service environment, the force after fracture decreases and then increases slightly, which is attributed to the force generated by the gradual fracture of some unbroken fibers.

**Figure 14:**
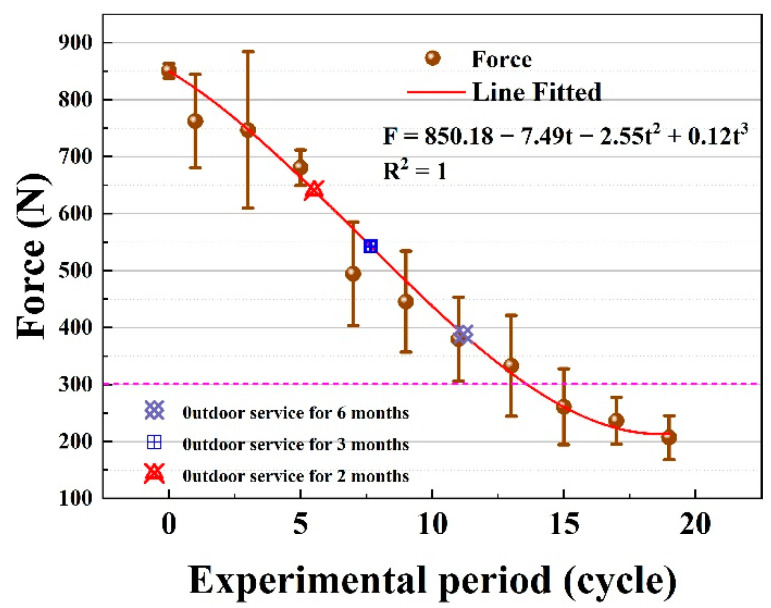
Experimental and fitting results of the breaking strength of the UHMWPE fiber in the environmental spectrum and in the real service environment.

To analyze the accelerated ratio of the environmental spectrum, the breaking strength of the UHMWPE fiber interception net aging in the environmental spectrum for different cycles and in the real service environment for different times is compared in Figure 14. The variation in the breaking strength of UHMWPE is fitted and the service life is evaluated. It is seen that the breaking strength of UHMWPE after aging in the real service environment for 2 months is located in the position of about 5.5 cycles on the fitting curve of the environmental spectrum, and the acceleration ratio is calculated to be 5.45. The breaking strength of UHMWPE after aging in the real service environment for 3 months locates in the position of about 7.5 cycles on the fitting curve of the environmental spectrum, and the acceleration ratio is calculated to be 6. For that in the real service environment for 6 months, the same breaking strength is located in the position of about 11 cycles on the fitting curve of the environmental spectrum, and the acceleration ratio is calculated to be 8. This reveals that the acceleration ratio increases with the elongation of aging time, which may be caused by the cross-linking and degradation of macromolecular chains in the material. When the cross-linking effect is greater than the chain fracture effect, the decrease in mechanical properties of the material is alleviated. 

## 4. Discussion

The environmental factors that affect the service life of the UHMWPE fiber interception net include temperature, humidity, light, chemical, and biological erosion [29]. To obtain the environmental spectrum that can be used to simulate the real service environment, the aging characteristics of UHMWPE fiber in individual salt spray, hygrothermal, and UV environments are analyzed. Ultraviolet light in natural light is located in the most deleterious radiation range for organic compounds: 295–385 nm [15,30,31], so it is regarded and proved as the most critical factor for failure of the UHMWPE fiber. Doğon [32] reported the controlling step that induces the degradation by UV. It is suggested that, after the molecular bond strengths’ energy limit surpasses UV energy absorption, degradation takes place through the free radical formation. Molecular bond strengths were summarized as C–C (320–720 kJ/mol), C–H (420–560  kJ/mol), and C–O (1000  kJ/mol) in degradation-based studies [33]. For the UV lamp with an energy range of 443–498 kJ/mol, it is capable to cause the rupture of the molecular nod structure of C–C and C–H bonds. Therefore, for UHMWPE, UV can cause the breakage of chemical bonds of polymer materials. The C-H and C-C bonds in the UHMWPE fiber absorb the ultraviolet radiation energy to form excited states and produce energy level transitions. The generated free radicals will continue to react with oxygen or water to form oxidized polymer free groups with higher reaction activity [34]. It also leads to the fracture and degradation of the fiber macromolecular chain, and ultimately reduces the breaking strength of UHMWPE (Figure 9c). In hot and humid environments where there is a lower oxygen concentration, fewer chain breaks are produced by the interaction of oxygen and free radicals in the molecular chain, so the attenuation of breaking strength is less obvious. However, the transport of water through microcracks or other forms of microdamage, such as pores or small channels, still present in the material and the micro-damages can be expanded by water [35]. The salt spray environment contains not only water molecules, but also inorganic salt ions that can erode the UHMWPE fiber, so salt spray aging has a more significant impact on breaking strength than the hygrothermal environment, as shown in Figure 9a.

**Figure 15:**
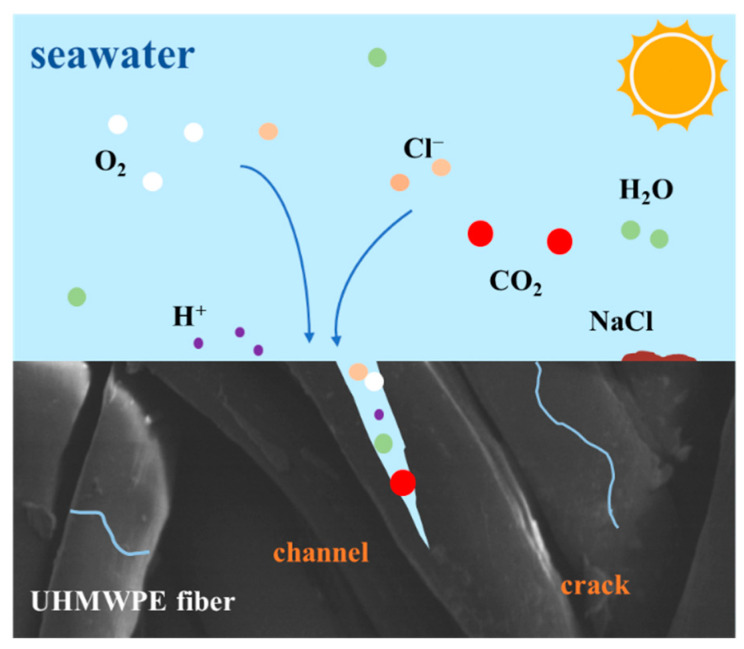
Schematic diagram of the aging process of the UHMWPE fiber in the real service environment.

According to the experimental results of aging in the individual salt spray, hygrothermal, and UV environments, the environmental spectrum is designed considering the influence of multiple factors existing in the real service environment. The UHMWPE fiber interception net is exposed to the interaction of light, heat, water, and salt spray in the actual marine environment, as schematically shown in Figure 15. UV irradiation can promote microcracks and pores in the UHMWPE fiber structure. The diffusion of water molecules and ions in microcracks, interfaces, and fiber cracks will lead to crack propagation and induce new cracks. At the same time, the long-term irradiation results in the increase in ambient temperature, and water molecule diffusion is accelerated. The hydrogen ions in water act as a catalyst of polymer oxygenation and polymerization, facilitating the degradation of the fiber.

## 5. Conclusions

To investigate the performance of the UHMWPE fiber interception net in the marine environment, the material is aged in salt spray, hygrothermal, and ultraviolet environments; a designed environmental spectrum; and the real service environment. The conclusions are as follows:

(1) The breaking strength deterioration in UV is more significant than that in the salt spray environment owing to changes in polymer chemical structure caused by chain breaking under light.
(2) After hygrothermal aging, some fine fibers bond into clumps, which lowers the attenuation of breaking strength.
(3) The environmental spectrum is designed according to the real environmental factors affecting the aging property. The degradation mechanism after aging in the environmental spectrum is the same to that in the real service environment.
(4) The acceleration ratio of the environmental spectrum to the real service environment increases with the elongation of aging time, which may be caused by the cross-linking and degradation of macromolecular chains in the material.

## Data Availability

The raw/processed data required to reproduce these findings can be obtained by contacting the corresponding author.
